# Clinical Outcomes and Cytokine Profile of Standard and Short Implant-supported Prostheses in Diabetics Treated for Periodontal Disease: A 5-year Study

**DOI:** 10.3290/j.ohpd.b5866861

**Published:** 2024-12-03

**Authors:** Abdulaziz A. AlHelal

**Affiliations:** a Abdulaziz A. AlHelal Associate Professor, Prosthetic Dental Science Department, College of Dentistry, King Saud University, Riyadh, Saudi Arabia.

**Keywords:** bone loss, inflammation, RANKL, short implant, type 2 diabetes

## Abstract

**Purpose:**

The present cross-sectional study aimed to assess the clinico-radiographic parameters as well as salivary levels of receptor activator of nuclear factor kappa-Β ligand (RANKL), osteoprotegerin (OPG), interleukin (IL)-6, and tumor necrosis factor-alpha (TNF-α) around standard and short dental implants (SDIs)-supported fixed partial denture in partially dentate type-II diabetes mellitus (T2DM) patients treated for periodontitis.

**Materials and Methods:**

The study comprised 4 groups: group 1 included T2DM patients with standard implants (n = 20); group II included non-T2DM patients with standard implants (n = 20); group III included T2DM patients with SDIs (n = 20); and group IV included non-T2DM patients with SDIs (n = 20). Participants eligible for the study included medically diagnosed T2DM patients with glycated hemoglobin (HbA1c) levels ≥ 6.5%, and non-T2DM participants with HbA1c levels between 4.0% and 5.0%. All had undergone previous periodontal therapy and had at least one standard implant and one SDI in the posterior maxillary or mandibular region. Exclusions were subjects with systemic conditions other than T2DM, recent use of steroids or antimicrobials, pregnancy or lactation, edentulism, misaligned dentition, or alcohol/tobacco use. Treatment involved non-surgical periodontal therapy, implant placement, and prosthetic procedures, with assessments including clinical (plaque index [PI], bleeding on probing [BOP], probing depth [PD]), radiographic (crestal bone loss [CBL]) parameters, and salivary cytokine levels including RANKL, OPG, IL-6, and TNF-α.

**Results:**

The study groups, each comprising 20 participants, showed no significant differences in demographics, restoration type, T2DM duration, family history, body mass index, or brushing routine (p>0.05). At baseline and 5-year follow-up, T2DM participants exhibited poorer periodontal parameters compared to non-T2DM, with higher PI (baseline: 62.2 ± 5.8% vs 29.6 ± 3.7%; 5-year follow-up: 69.2 ± 6.1% vs 32.8 ± 3.8%), BOP (baseline: 30.5 ± 3.2% vs 18.2 ± 2.6%; 5-year follow-up: 35.5 ± 3.9% vs 20.5 ± 2.5%), PD (baseline: 5.5 ± 1.1 mm vs 3.1 ± 0.9 mm; 5-year follow-up: 4.2 ± 0.8 mm vs 2.4 ± 0.7 mm), and CBL (baseline: 4.4 ± 0.4 mm vs 2.0 ± 0.2 mm; 5-yearfollow-up: 4.9 ± 0.5 mm vs 2.3 ± 0.3 mm), regardless of implant type. Salivary cytokine levels (RANKL, OPG, IL-6, TNF-α) were consistently higher in T2DM groups than non-T2DM across both implant types. Participants with SDIs showed comparable clinico-radiographic outcomes and salivary levels of cytokines to standard implants.

**Conclusion:**

The application of SDI-supported rehabilitation in T2DM and non-diabetics showed comparable clinico-radiographic outcomes and salivary levels of cytokines to standard dental implants. Furthermore, T2DM patients exhibit poorer periodontal health and elevated inflammatory markers in patients with standard implants and SDIs.

The maintenance of peri-implant bone level plays a crucial role in determining the success of dental implants. Numerous factors, such as systemic inflammatory conditions including type-II diabetes mellitus (T2DM) and obesity, consumption of smokeless tobacco, smoking habits, and oral hygiene care, significantly contribute to the overall success of the procedure.^3,17,34,44^ Chronic hyperglycemia, in particular, is known to be a typical risk factor that leads to soft tissue inflammation and resorption of bone around implants and natural dentition.^37^ This can be attributed to the increased levels of accumulated glycation end products (AGEs) in the oral tissues and serum, which stimulate the production of proinflammatory cytokines and subsequently result in bone destruction around implants and natural dentition.^12,30^ Nevertheless, it is important to note that under ideal glycemic control, implants can successfully osseointegrate and yield predictable results over long periods, irrespective of whether the patients are diabetic or not.^10^

Recently, there has been an increased utilisation of short dental implants (SDIs) by dental implant professionals. The nomenclature “SDIs” is a matter of personal interpretation, lacking a universally agreed upon definition.^8^ Consequently, a consensus regarding its accurate delineation has yet to be reached.^10^ Some scholars have characterised SDIs as implantable devices with a length measuring 10 mm, whereas alternative healthcare professionals have designated them as “implants not exceeding 7 mm in length”.^18^ This particular variety of implants can be employed within regions characterised by insufficient bone volume, thereby eliminating the necessity to perform intricate surgical procedures, including bone augmentation, distraction osteogenesis, and sinus floor elevation.^7,38^ Concerning atrophic ridges in the posterior maxillary region, the utilisation of SDIs presents a favorable approach for the rehabilitation of areas with missing teeth. Additionally, this alternative therapeutic approach confers benefits to the individual, as it is comparatively cost-effective and reduces the duration of surgery.^35^ According to Esfahrood et al,^21^ the survival rate of SDIs is high when they are placed in maxillary posterior edentulous areas. Similarly, Renouard et al^45^ revealed a cumulative survival rate of 95% for SDIs in the severely resorbed maxilla. Additionally, when used to restore completely or partially edentulous mandibles with fixed or removable prostheses, SDIs demonstrated a 99% survival rate.^26^ Consequently, Grant et al^26^ concluded that SDI can be regarded as a promising treatment alternative to complex surgical procedures for addressing atrophied mandibular ridges.

In periodontitis, the receptor activator of nuclear factor kappa-Β ligand (RANKL) is increased, while osteoprotegerin (OPG) is decreased, leading to a higher RANKL:OPG ratio, associated with bone loss and disease progression.^16^ This imbalance contributes to osteoclast formation and subsequent bone loss in periodontitis, making the RANKL/OPG system a potential therapeutic target.^33^ Interleukin-6 (IL-6) plays a vital role in the initiation and acute phase of periodontitis, serving as a pro-inflammatory mediator triggered by pathogen-associated stimuli and other cytokines.^28^ It contributes to dysbiotic host responses, fostering local and systemic inflammation in periodontitis.^40^ IL-6 is also linked to bone homeostasis and is implicated in the risk and pathogenesis of periodontal disease.^52^ While some studies suggest a potential protective role, the majority emphasise its pro-inflammatory nature in the context of periodontitis.^4,41^ Periodontitis leads to increased tumor necrosis factor-alpha (TNF-α) levels in the gingival crevicular fluid, contributing to the degeneration of inflamed periodontal tissues.^11^ Elevated TNF-α levels are observed in patients with clinical indicators of periodontitis,^31^ suggesting its potential as a biomarker for diagnosis and management. Studies also support the use of salivary TNF-α levels as a biomarker for detecting periodontal diseases.^39,50^

The literature contains an extensive amount of research data concerning the long-term success of implants in patients who have periodontal health.^20^ However, there is a lack of understanding regarding the outcomes of implant therapy in patients who have compromised periodontal condition.^6^ For this reason, the importance of periodontal treatment for the remaining teeth before the placement of implants has been emphasised.^2^ Numerous studies exist that have assessed the short- and long-term outcomes of implants in patients with periodontitis.^24,47^ Until now, follow-up studies have been lacking regarding the clinical and radiographic peri-implant outcomes of SDIs placed in patients who have been treated for periodontitis, resulting in a lack of clarity in this area. Recent evidence regarding the clinical and radiographic peri-implant status in various levels of glycemia indicates that these factors are considerably compromised in individuals with elevated glycemic levels in comparison to those with well-regulated glycemic control.^26,40^ Nonetheless, data are scarce concerning the outcomes of SDIs among patients with different glycemic levels. The authors hypothesise that patients with elevated glycemic levels will demonstrate less favorable clinico-radiographic peri-implant parameters, specifically plaque index (PI), bleeding on probing (BOP), probing depth (PD), and crestal bone loss (CBL) around SDIs compared to non-diabetic patients. Hence, the present study aimed to assess the clinico-radiographic parameters as well as salivary levels of RANKL, OPG, IL-6, and TNF-α around standard implants- and SDI-supported fixed partial dentures in partially dentate type-2 diabetes mellitus (T2DM) patients treated for periodontitis.

## MATERIALS AND METHODS

### Study Protocol and Ethical Considerations

The current single-centered study was conducted in accordance with the fundamental principles of the Declaration of Helsinki, encompassing human subjects, and followed the Strengthening the Reporting of Observational Studies in Epidemiology (STROBE) recommendations. The study protocol was reviewed and approved by the research and ethics committee of the Specialist Dental Practice and Research Center in Riyadh (UDCRC-038-017). All individuals who volunteered for the study were obligated to sign a consent form in both Arabic and English, using clear and concise language. Participants were duly informed that they possessed the right to withdraw their participation at any stage of the study, without incurring any penalties.

### Participant Eligibility Criteria

The inclusion criteria of the participants comprised: (i) medically diagnosed patients with T2DM having glycated hemoglobin (HbA1c) levels ≥ 6.5%;33 (ii) non-T2DM participants with HbA1c levels ranging from 4.0% to 5.0%;15 (iii) patients who had previously undergone periodontal therapy for the treatment of periodontitis; and (iv) patients having a minimum of one standard implant and one SDI in the posterior maxillary or mandibular region.

The exclusion criteria were subjects who: (i) self-reported systemic conditions (except T2DM) including renal, hepatic, cardiovascular diseases, or acquired immune deficiency syndrome; (ii) consumed steroids or antimicrobial agents over the previous 6 months; (iii) were pregnant or lactating women; (iv) were edentulous individuals; (v) had misaligned dentition; and (vi) consumed alcohol or tobacco.

### Research Groups

The study consisted of four groups. Group I included T2DM patients with standard implants; group II consisted of non-T2DM individuals with standard implants; group III comprised T2DM patients with SDIs; and group IV included non-T2DM participants with SDIs.

### Power Calculation

The sample size was estimated based on data from a previous pilot study, which involved a group of 4 patients, each of whom had 4 peri-implant locations. This accounted for a total of 16 peri-implant locations in each group. The final sample size was calculated, taking into account a 5% alpha (significance level), 95% power (1-beta), and a standard deviation (SD) of 1.2. In total, 20 peri-implant locations in individual research groups were determined to be sufficient for assessing a minimum difference of 1 mm in CAL between the control and experimental groups.

### Calibration Training

The calibration procedure was executed prior to study commencement, following the methodology as described elsewhere.^13^ A measurement of 0.3 mm for PD and 0.3 mm for CAL was determined for the inter-examiner variability. In the first examiner, the intra-examiner mean SE variability was 0.1 mm for PD and 0.1 mm for CAL. Meanwhile, the second examiner exhibited a mean SE variability of 0.20 mm and 0.22 mm for PD and CAL, respectively. The periodontal parameters were documented dichotomously, encompassing plaque accumulation, gingival bleeding, BOP, and suppuration, which were assessed through two separate evaluations utilising the k-light test (p < 0.05), considering the element of chance agreement. The inter-observer agreement levels varied from 0.85 to 0.95, while the intra-observer agreement fell within the range of 0.80 to 0.96 for the first examiner and 0.80 to 0.87 for the second examiner.

### Periodontal Intervention

All patients underwent non-surgical treatment for their periodontal condition, which involved subgingival scaling and root planing (SRP) performed under local anesthesia. This procedure was specifically targeted at tooth surfaces that exhibited a PD > 4 mm and continuous BOP. The patients were also instructed in strict oral hygiene practices and given reinforcement in this area. The SRP was carried out in two sessions 1 week apart at baseline. In addition, any supragingival calculus that was present around implants was removed using plastic curettes. Periodontal treatment was scheduled for regular follow-up visits at 3-month intervals and continued for 5 years. Hopeless teeth were extracted, and subgingival periodontal treatment was provided for the remaining teeth where required. After the periodontal treatment, amoxicillin (500 mg)/TID was prescribed for 1 week for preventing infections during the early healing phase.

### Implant Placement and Prosthetic Procedure

An experienced and trained oral surgeon (AA) performed all surgeries while employing local anesthesia. Pre-operatively, every patient was given a prophylactic antibiotic dose of 1000 mg of amoxicillin, commencing the evening before the surgical intervention. Subsequently, a daily dosage of 1500 mg amoxicillin was prescribed for 1 week post-operatively. In cases where patients had a penicillin allergy, an alternate pre-operative and post-operative antibiotic, clindamycin 2000 mg daily, was administered. Moreover, an analgesic (ibuprofen, 600 mg as required, every 6 to 8 h) was also given. Participants were further instructed to perform oral rinsing twice per day utilising a solution of 0.2% chlorhexidine digluconate for two weeks, starting on the day before surgery. Full-thickness mucoperiosteal flaps were reflected through a midline crestal incision. The preparation of the implant osteotomy sites followed a standardised drilling sequence, as previously detailed in the literature.^9^ To minimise the potential for injury to the inferior alveolar or maxillary sinus, a precautionary measure was taken: Adjustable rubber stops were utilised to ensure that the drills were positioned at a distance of a minimum of 1 mm below the radiographic working length above the maxillary sinus and mandibular canal. In cases where maxillary/mandibular premolars and/or molars were absent, up to two SDIs (length: 6 mm; diameter: 4 mm [OsseoSpeed, DENTSPLY Implants; Molndal, Sweden]) were inserted. These implants were placed at the level of the bone and remained submerged for a period of three to four months. Subsequently, the implants were loaded. Following a healing abutment connection, screw-retained porcelain-fused-to-metal fixed dental prostheses were provided after a period of eight to ten weeks. Regular maintenance care was implemented for all patients, with biannual appointments for full-mouth mechanical plaque and calculus debridement using handheld ultrasonic scalers (ART-M1 Magnetostrictive Ultrasonic Scaler Unit; Cary, NC, USA). Oral hygiene instructions were reinforced during each recall appointment for individuals in both groups.

### Research Questionnaire

Under the supervision of a clinician, all study participants completed a structured questionnaire consisting of the following information: (i) demographics, including age and sex; (ii) duration of the implant in service; (iii) brushing habits; (iv) cause of missing tooth/teeth; (v) duration of T2DM; (vi) family history of T2DM; (vii) mean body mass index [BMI] in kg/m^2^ [min – max]; and (viii) HbA1c levels (mean% ± SD)].

### Assessment of HbA1c Levels

The examination of serum glycemic status involved a thorough review of medical records, along with a recent assessment of HbA1c levels to gauge the three-month profile. The verification of non-diabetic status was confirmed through the HbA1c test. Participants were divided into two groups based on the classification outlined by the American Diabetes Association.^33^ Chair-side HbA1c levels were determined for both groups using an HbA1c analyser kit (Quo-Test, EKF Diagnostics; Magdeburg, Germany). Morning venipuncture from the antecubital vein was performed to draw serum samples, which were collected in vacutainer tubes with a gel separator, and in heparinised tubes for HbA1c measurements. The samples were incubated for 30 min and then centrifuged at 2000 rpm at 4°C for 15 min.

Patients in T2DM groups received guidance from their medical physicians on managing blood glucose levels through dietary recommendations. Moreover, they were specifically advised to undergo treatment with anti-diabetic medications and were also counseled to adhere to dietary control measures.

### Assessment of Peri-Implant Clinical and Radiographic Parameters 

A calibrated and trained investigator (AA) conducted all clinico-radiographic assessments. The intra-examiner reliability, with an overall kappa of 0.85, reflected high consistency. Peri-implant BOP36 and PD14 were assessed at six locations per implant, including mesio-palatal/lingual, mid-palatal/lingual, disto-palatal/lingual, disto-buccal, mid-buccal, and mesio-buccal. Peri-implant PD was measured to the nearest millimeter using a graded probe (Hu-Friedy; Chicago, IL, USA).^29^ In all study groups, intra-oral digital bitewing radiographs were taken for each implant, employing a standardised technique with a film holder as a guide for x-ray beams (Belmont ACURAY 071A Intra Oral X-Ray System; Hudson, FL, USA). CBL was defined as the linear distance from the implant-abutment junction to the most coronal part of the alveolar crest.19,40 CBL was recorded in millimeters using Scion Image software (Scion; Frederick, MD, USA).

### Collection of Whole Saliva Sample

Resting whole-saliva samples were collected in the morning, after a minimum fasting period of two h (between 9:00 a.m. and 11:00 a.m.), utilising the Salivette system (Sarstedt; Nümbrecht, Germany). In brief, the patient extracted the swab from the Salivette and inserted it into the oral cavity. After ca 5 min, the patient reintroduced the swab containing the absorbed saliva back into the Salivette. The obtained saliva specimens were then subjected to centrifugation for 120 s at 1000 x g, and the resultant supernatants were carefully transferred into Eppendorf tubes. These tubes were subsequently stored at a temperature of -80°C until the day of performing RANKL, OPG, IL-6, and TNF-α assays. Subsequently, these saliva samples were thoroughly thawed, subjected to vortex mixing, and subsequently centrifuged at a force of 1500x for 15 min. The purpose of this step was to eliminate mucins and other particulate matter that could potentially interfere with the binding of antibodies and subsequently influence the results of RANKL, OPG, IL-6, and TNF-α tests.

### Assessment of Salivary Cytokine Levels

Whole salivary levels of RANKL, OPG, IL-6, and TNF-α were quantified using an enzyme-linked immunosorbent assay (ELISA). Human RANKL (R & D Systems; Minneapolis, MN, USA), OPG (R&D Systems), IL-6 (human interleukin-6 Quantikine ELISA Kit, R&D Systems), and TNF-α (human TNF-alpha Quantikine ELISA Kit; Minneapolis, MN, USA) kits were employed following the instructions provided by the manufacturers. In brief, as described elsewhere,^1,48^ the construction of a standard curve utilised RANKL, OPG, IL-6, and TNF-α standards that were included with the kits, and the protein concentration was calculated based on this curve. A total of 100 μl of diluted standards, along with the samples, were then distributed in duplicate into the wells that were coated with a specific protein antibody. The plates were incubated for 60 min per day. Subsequently, a wash solution was used to clean the plates three times. Following this, a 100-μl solution of conjugate was added, and the plates were incubated at room temperature for an additional 120 min. The wells were once again cleaned with the wash solution three times. Then, a total of 100 μl of substrate solution was added. The plates were incubated at room temperature for 20 min, after which 50 μl of stop solution was added to halt color development. Absorbance was measured by observing the plate at a wavelength of 450 nm using a spectrophotometer.

### Statistical Analysis

The statistical software SPSS (Statistics 28.0.1.1 Windows, IBM; Armonk, NY, USA) was utilised to conduct the statistical analysis. The peri-implant clinico-radiographic parameters were presented in the form of means and percentages. To evaluate the normal distribution of the data, the Kolmogorov-Smirnov test was employed. For categorical data sets, Pearson’s chi-squared test was utilised, while the Kruskal-Wallis test was employed to compare the means between different groups. To perform multiple comparisons, the Bonferroni test was applied. The significance level was set at p < 0.05.

## RESULTS

### Primary Features of Study Participants

Table 1 depicts the general characteristics of the study groups’ participants including their demographics, restoration type, reason for missing teeth, and brushing routine. Each group consisted of 20 participants. Gender distribution showed no statistically significant difference among groups (p = 0.09), and mean ages ranged from 51 to 53 years, with no statistically significant variation (p = 0.11). There were also no statistically significant differences in the total number of standard implants vs SDIs among the groups (p = 0.21). T2DM duration and family history of T2DM exhibited no statistically significant differences among groups (p = 0.10 and p = 0.22, respectively). Mean BMI also showed no statistically significant differences (p = 0.18). Similarly, restoration type (cemented/screw-retained) did not statistically significantly differ among the groups (p = 0.39). The most common reason for missing teeth was caries, followed by periodontal disease and trauma. Brushing routines and frequency of dental visits also showed no statistically significant differences among the groups (p = 0.45 and p = 0.16, respectively).

**Table 1:** Primary features of the study participants

Study parameters	Group I (T2DM + standard implants)	Group II (non-T2DM + standard implants)	Group III (T2DM + SDIs)	Group IV (non-T2DM + SDIs)	Significance (p-value)
Participants (n)	20	20	20	20	NA
Gender (n = male/female)	08/12	09/11	10/10	08/12	0.09
Mean age in years (mean ± SD)	51 ± 4.5	53 ± 3.6	53 ± 3.8	52 ± 4.1	0.11
Total implants (n = standard/SDI)	13/11	11/12	11/10	12/12	0.21
Functional implant duration (months)	71.4 ± 8.5	77.8 ± 10.9	76.2 ± 9.8	74.5 ± 9.3	NA
Diabetes duration (years [mean ± SD])	8.9 ± 3.2	NA	9.1 ± 3.8	NA	0.10
Family history of T2DM (n)	11	2	13	3	0.22
HbA1c levels (mean ± SD)	8.8 ± 1.3	NA	7.9 ± 1.2	NA	NA
Mean BMI in Kg/m^2^ (min – max)	36.8 (30.2 – 41.5)	31.5 (27.5 – 35.7)	33.7 (29.1 – 36.9)	32.8 (28.5 – 35.2)	0.18
Restoration type Cemented (n) Screw-retained (n)	4 20	4 19	5 21	5 20	0.39
Missing tooth/teeth reason (%) Dental caries Periodontal disease Trauma	70 30 0	75 25 0	70 24 1	80 20 0	0.12
Brushing routine (%) Once daily Twice daily	17 3	16 4	18 2	17 3	0.45
Frequency of dental visits (n)	2	5	2	7	0.16


### Clinical and Radiographic Parameters

Table 2 presents the clinico-radiographic parameter values at baseline and 5-year follow-up visits for participants in different study groups. At baseline, statistically significant differences were observed among groups for all parameters (p < 0.05). Group I participants (T2DM with standard implants) had higher PI (62.2 ± 5.8%), greater BOP (30.5 ± 3.2%), deeper PD (5.5 ± 1.1 mm), and more significant CBL (4.4 ± 0.4 mm) compared to group II participants (PI: 29.6 ± 3.7%; BOP: 18.2 ± 2.6%; PD: 3.1 ± 0.9 mm; CBL: 2.0 ± 0.2 mm). A similar pattern was observed between T2DM and non-T2DM groups with SDIs.

**Table 2:** Values of clinico-radiographic parameters in different study group participants at baseline and 5-year follow-up

Peri-implant parameters	Study groups	Baseline	5-year follow-up
Plaque index (%)	T2DM + standard implant Non-T2DM + standard implant T2DM + SDI Non-T2DM + SDI	62.2 ± 5.8^a^ 29.6 ± 3.7^b^ 61.1 ± 5.2 ^a^ 30.1 ± 3.5^b^	69.2 ± 6.1^a^ 32.8 ± 3.8^b^ 71.5 ± 6.3^a^ 34.7 ± 3.5^b^
Bleeding on probing (%)	T2DM + standard implant Non-T2DM + standard implant T2DM + SDI Non-T2DM + SDI	30.5 ± 3.2^a^ 18.2 ± 2.6^b^ 32.8 ± 3.6^a^ 19.8 ± 2.9^b^	35.5 ± 3.9^a^ 20.5 ± 2.5^b^ 37.1 ± 4.1^a^ 22.8 ± 2.8^b^
Probing depth (mm)	T2DM + standard implant Non-T2DM + standard implant T2DM + SDI Non-T2DM + SDI	5.5 ± 1.1^a^ 3.1 ± 0.9^b^ 5.8 ± 1.3^a^ 3.3 ± 1.9^b^	4.2 ± 0.8^a^ 2.4 ± 0.7^b^ 4.4 ± 1.0^a^ 2.8 ± 0.9^b^
Crestal bone loss (mm)	T2DM + standard implant Non-T2DM + standard implant T2DM + SDI Non-T2DM + SDI	4.4 ± 0.4^a^ 2.0 ± 0.2^b^ 4.6 ± 0.5^a^ 2.2 ± 0.4^b^	4.9 ± 0.5^a^ 2.3 ± 0.3^b^ 5.1 ± 0.7^a^ 2.5 ± 0.5^b^
Different superscript letters indicate statistically significant difference (p < 0.05).

At the 5-year follow-up, these differences persisted, with T2DM groups generally exhibiting poorer periodontal parameters (PI: 69.2 ± 6.1%; BOP: 35.5 ± 3.9%; PD: 4.2 ± 0.8 mm; CBL: 4.9 ± 0.5 mm) compared to non-T2DM groups across both implant types (PI: 32.8 ± 3.8%; BOP: 20.5 ± 2.5%; PD: 2.4 ± 0.7 mm; CBL: 2.3 ± 0.3 mm). A similar pattern was observed between T2DM and non-T2DM groups with SDIs.

### Salivary Cytokine Levels

Table 3 displays the salivary levels of peri-implant cytokines RANKL, OPG, IL-6, and TNF-α among participants in different study groups. T2DM groups (groups I and III) consistently exhibited higher levels of these cytokines compared to their non-T2DM counterparts across both standard implants and SDIs. Specifically, participants with T2DM and standard implants demonstrated elevated levels of RANKL, OPG, IL-6, and TNF-α compared to non-T2DM individuals with standard implants. A similar pattern was observed between T2DM and non-T2DM groups with SDIs.

**Table 3:** Salivary levels of peri-implant RANKL/OPG, IL-6, and TNF-α in different study group participants

Peri-implant salivary cytokines	T2DM + standard implant	Non-T2DM + standard implant	T2DM + SDI	Non-T2DM + SDI
RANKL (pg/ml)	38.6 ± 10.1^a^	23.2 ± 5.5^aa^	38.9 ± 10.6^a^	23.1 ± 5.4^a^
OPG (pg/ml)	32.5 ± 9.5^bb^	21.8 ± 3.5^b^	33.0 ± 10.1^b^	22.5 ± 4.2^b^
IL-6 (pg/ml)	26.1 ± 18.6^a^	11.2 ± 2.3^a^	27.2 ± 17.2^a^	12.2 ± 3.0^a^
TNF-α (pg/ml)	24.6 ± 9.2^b^	12.9 ± 2.5^b^	26.2 ± 10.1^b^	14.0 ± 4.2^b^
Different superscript letters indicate statistically significant difference (p < 0.05).

### DISCUSSION

The present study aimed to assess the clinico-radiographic parameters and salivary levels of RANKL, OPG, IL-6, and TNF-α around standard implant- and SDI-supported fixed partial dentures in partially dentate T2DM patients treated for periodontitis. The findings of the clinical and radiographic parameters revealed statistically significant differences at baseline and 5-year follow-up, with T2DM participants consistently exhibiting poorer periodontal health compared to non-T2DM counterparts across both standard implants and SDIs. Additionally, salivary cytokine levels indicated elevated inflammatory markers in T2DM groups (groups I and III), suggesting a potential link between T2DM and adverse peri-implant outcomes. Moreover, standard implants and SDIs demonstrated comparable outcomes associated with clinico-radiographic periodontal parameters (PI, BOP, PD, and CBL) as well as salivary levels of cytokines (RANKL, OPG, IL-6, and TNF-α).

The high degree of inflammation surrounding peri-implant tissues in patients with elevated glycemic levels may be attributed to multiple biomolecular factors. One potential mechanism could be the excessive accumulation of AGEs, which are formed through non-enzymatic glycosylation of various proteins in the serum of individuals with diabetes mellitus.^51^ The elevated levels of AGEs further stimulate the production of receptors for AGEs.^27^ This interaction ultimately gives rise to the generation of various types of detrimental proinflammatory cytokines, including IL-6 and collagenases (matrix metalloproteinase), which are synthesised by periodontal and peri-implant gingival fibroblasts. These cytokines play a crucial role in the inflammation and destruction of both periodontal and peri-implant tissue.^9^

Furthermore, it is important to note that the mean BOP was also elevated in patients who had T2DM. These findings align with the results of Gomez-Moreno et al,^25^ who demonstrated a heightened tendency for bleeding in patients with higher levels of HbA1c. Another recent cross-sectional study^43^ provided further evidence that both prediabetic and diabetic conditions are correlated with an increased inclination for BOP. Additionally, it is worth mentioning that the average peri-implant PD around SDIs was statistically significantly higher in patients with T2DM compared to non-diabetics. This implies that the mean PD measurements were not sufficiently deep to be considered pathological in patients with T2DM.

The clinical and radiographic periodontal parameters, namely PD and CBL, respectively, were compromised in the study groups in the current investigation. It is worth noting that individuals with a history of chronic periodontitis have been recorded to exhibit an increased susceptibility to peri-implant inflammation.^46^ Preliminary studies have indicated that the microbiota surrounding failing dental implants and periodontally affected teeth possess similar bacterial compositions, characterised by a substantial presence of gram-negative anaerobic rods.^32^ In this particular study, the analysis of the microbiological profile among patients with peri-implant inflammation and periodontal infection was not conducted. However, future studies should investigate the microbiological profile in individuals with peri-implant inflammation and different levels of periodontitis among diabetic and prediabetic subjects to yield robust conclusions.

Another noteworthy discovery in the current investigation is the observation that individuals in the T2DM cohorts exhibited elevated average BMI values ranging from 33.7 to 36.8 kg/m^2^. Recent data from both cross-sectional and longitudinal studies suggest that increased body weight serves as a significant systemic risk factor for the inflammation of both periodontal and peri-implant tissues.^5,49^ A recent study by Vohra et al^49^ found that patients with high BMI are more susceptible to heightened inflammation in the peri-implant region and subsequent loss of crestal bone when compared to individuals of normal weight. It is speculated in this particular case that the presence of oxidative stress, which arises as a result of being overweight or obese,^23^ may contribute to the heightened inflammation in the peri-implant region in individuals with increased body weight, thereby further compromising clinical and radiographic measures of peri-implant health in diabetic patients. Additional investigation is necessary to validate this hypothesis.

Cytokine levels in saliva are generally higher compared to peri-implant crevicular fluid (PICF) and saliva.^22,42^ However, some specific cytokines like IL-1β are elevated in PICF of peri-implantitis sites compared to healthy sites.^22^ Mean levels of all cytokines tested (TNFα, IFNγ, IL-10, IL-12p70, IL-13, IL-1β, IL-2, IL-4, IL-6, IL-8) were higher in saliva compared to PICF.42 In peri-implantitis patients, IL-1β levels were statistically significantly higher in deep PICF sites compared to mucositis sites.^22^ Salivary IL-8 and IL-12 levels were significantly higher in peri-implantitis patients compared to mucositis patients.^22^ Salivary cytokine levels showed strong positive intercorrelations with each other, more so than cytokines in plasma.^42^ Some plasma cytokine levels (e.g., IFNγ) correlated well with levels of certain salivary cytokines (e.g. TNFα, IL-12p70, IL-2, IL-10).^42^ In summary, while overall cytokine levels are higher in saliva, specific cytokines like IL-1β are locally elevated in the PICF of diseased peri-implant sites, suggesting their role in peri-implant inflammation. Saliva may represent systemic inflammatory status better than PICF.

The present study found that the clinico-radiographic periodontal outcomes as well as the levels of salivary cytokines around SDIs are comparable to those of standard dental implants, signifying that SDIs can be a viable alternative with similar effectiveness. The significance of this comparability lies in the potential for SDIs to offer comparable stability, osseointegration, and inflammatory response to standard implants. This can expand the treatment options for patients with reduced bone volume and anatomical limitations, providing a reliable and effective solution for implant-supported restorations. Additionally, it may indicate that the use of SDIs does not compromise the peri-implant tissue health and inflammatory response, further supporting their clinical utility and long-term success.

## CONCLUSION

The application of SDI-supported rehabilitation in T2DM and non-diabetic periodontitis patients showed comparable clinico-radiographic outcomes and salivary levels of cytokines to standard dental implants. Furthermore, T2DM patients exhibit poorer periodontal health and elevated inflammatory markers in patients with standard implants and SDIs.
